# Association of four gene polymorphisms in Chinese Guangxi population with diabetic retinopathy in type 2 diabetic patients

**DOI:** 10.1186/s12886-021-02146-4

**Published:** 2021-10-27

**Authors:** He Jin, Dongdong Jiang, Zhixiang Ding, Yu Xiong, Xinsheng Zeng, Miaoyun Liao, Liu Zheng, Binbin Yang

**Affiliations:** 1 Affiliated Hospital of Guilin Medical University, Guilin Medical University, Guilin, 541001 China; 2 Nanxishan Hospital of Guangxi Zhuang Autonomous Region, Guilin Medical University, Guilin, 541001 China

**Keywords:** Diabetic retinopathy, Single nucleotide polymorphisms, Type 2 diabetes mellitus, KASP genotyping assays

## Abstract

**Background:**

Diabetic retinopathy (DR) is one of the most common chronic microvascular complications of diabetes. Many studies have suggested that genetic factors are important in the context of DR. This study evaluated the associations of GWAS (Genome-wide association study) -identified DR-associated SNPs in a Chinese population in Guangxi Province with type 2 diabetes mellitus (T2DM).

**Methods:**

A total of 386 hospitalized T2DM patients without proliferative diabetic retinopathy (PDR) and 316 hospitalized T2DM patients with PDR were included in this case–control study. Four tag SNPs, including rs1800896 in the IL-10 gene, rs2010963 in the VEGFA gene, rs2070600 in the RAGE gene and rs2910164 in the miR-146a gene, were examined using KASP (kompetitive allele specific PCR) genotyping assays.

**Results:**

There were no significant differences in the genotype or allele frequencies of the miR-146a polymorphism (rs2910164) between subjects with PDR and those without DR. The TC genotype of rs1800896 was determined to be associated with an increased risk of PDR (the odds ratio (OR) was 2.366, with a 95% confidence interval (CI) ranging from 1.144 to 4.894). The CG genotypes of rs2010963 was associated with an decreased risk of PDR (the OR was 0.588, with a 95% CI ranging from 0.366 to 0.946). Regarding rs2070600, 2 genotypes (TT and CT) were associated with a decreased risk of PDR (the OR of the TT genotype was 0.180, with a 95% CI ranging from 0.037 to 0.872, and the OR of the CT genotype was 0.448, with a 95% CI ranging from 0.266 to 0.753).

**Conclusions:**

The rs1800896 polymorphisms in the IL-10 gene, rs2010963 in the VEGFA gene and rs2070600 in the RAGE gene are associated with the risk of PDR in the Han Chinese population of Guangxi Province. Our findings provide suggestive evidence that these polymorphisms may be involved in the pathogenesis of PDR and should be investigated further.

## Introduction

Diabetes is an endocrine disease that severely impacts human health, and its disability and fatality rates are second only to those of cardiovascular and cerebrovascular diseases and cancer [1]. It is estimated that the percentage of people with diabetes worldwide will reach 4.4% by 2030 [2]. It has been recognized that diabetes is a main source of morbidity and mortality given its related acute and chronic side effects. Diabetic retinopathy (DR) is one of the most common chronic microvascular side effects of diabetes [3]. With the incidence of diabetes increasing worldwide, the incidence of DR is expected to increase to alarming levels [4]. Furthermore, DR is the main cause of blindness in diabetic patients [3].

Diabetes duration, poor glycaemic control and hypertension are known as the primary risk factors related to the progression of DR [4]. However, clinical observation has revealed that some patients with poorly controlled or long-lasting diabetes do not develop retinopathy, whereas others, even those with relatively good glycaemic control, eventually develop advanced retinopathy [5]. These clinical observations suggest that other factors are involved in the development of DR. Many studies suggest that genetic factors are important in the context of DR, that DR exhibits a complicated inheritance mode and that genetic relationship studies are useful for the identification of the genetic elements impacting the pathogenesis of DR [6]. According to this information, genetic elements exert an effect on the development of DR [7]. It has been estimated that the heritability of DR is approximately 25% [8]. Through an increased understanding of the genetic foundation of DR, the hidden pathophysiological mechanisms governing its development can be identified. These genetic data may also be useful for the risk profiling of DR among patients suffering from diabetes, thereby promoting its early treatment and administration. Robust relationships of DR-susceptibility variants may make them ideal genetic markers to enhance the prediction of DR via traditional clinical predictors and thus achieve more precise risk stratification.

Genome-wide association studies (GWASs) represent a selective strategy for the detection of new genetic loci related to DR, and some GWASs have been performed using different ethnic groups to identify novel genetic variants related to DR susceptibility in diabetes mellitus cohorts [9–12]. Single nucleotide polymorphisms (SNPs) are deoxyribonucleic acid (DNA) sequences that commonly differ in populations [13]. These changes in DNA sequences may affect gene expression if they occur in putative regulatory regions [13]. In several previous reports, some GWAS-identified SNPs were found to be highly associated with DR [6, 14–16].

In our previous study, we analysed the association of 20 SNPs selected from 11 DR-associated factors, which were reported in previous reports [6, 14, 16–24]. These 11 DR-associated factors were Interleukin-10 (IL-10), Vascular endothelial growth factor (VEGF), Receptor for advanced glycation end product (RAGE), microRNA-146a (miR-146a), Intercellular adhesion molecule-1 (ICAM-1), complement factor H (CFH), Transforming growth factor-β1 (TGF-β1), Endothelin 1 (EDN1), Osteoprotegerin (OPG), C-reactive protein (CRP), Tumor necrosis factor-α (TNF-α). The causal relationship between inflammation and angiogenesis in PDR is widely accepted [25]. And in the Petrovič’s study [26], they summarized the IL-10, VEGF and RAGE were the candidate genes for proliferative diabetic retinopathy (PDR). The miR-146a was also associated with microvascular complications [16]. And those four DR-associated factors were across varying diseases in different ethnicities [16, 18]. According to the previous reports [6, 16, 27, 28], we selected 4 SNPs of those 4 factors, which were identified in the GWAS. And whether these four DR-associated SNPs influence PDR in the Han Chinese population of Guangxi Province has not been examined in a systematic way. Therefore, this study primarily aims to assess the relationships of GWAS-recognized DR-related SNPs in this population with type 2 diabetes mellitus (T2DM).

## Materials and approaches

### Research population

The research protocol was examined by the Research Ethics Committee of the Affiliated Hospital of Guilin Medical University and adhered to the principles of the Declaration of Helsinki. All participants gave written informed consent before their enrolment. A total of 386 T2DM patients without diabetic retinopathy (DR) and 316 T2DM patients with proliferative diabetic retinopathy (PDR) were included in this case–control study. All patients’ data were collected from the endocrinology department and ophthalmology department and were included in the study in the enrolment. All patients lived in Guangxi Province and were Han Chinese. The diagnosis of type 2 diabetes was made on the basis of the American Diabetes Association criteria [29]. All patients received ophthalmic examinations such as best-modified visual acuity, intraocular pressure, slit lamp, and dilated fundus examinations, in the Department of Ophthalmology, Affiliated Hospital of Guilin Medical University. PDR was defined as having eyes with definite neovascularization and/or vitreous/preretinal haemorrhages. Patient and medical data, including age, sex, age at diabetes diagnosis, presence of arterial hypertension, application of medication or insulin, and other comorbidities, were collected using a questionnaire. Individuals with peripheral vascular diseases, coronary artery diseases, acute infection, history of any thrombotic event, or any other ocular disorders were excluded.

### Genotyping

Whole-blood specimens from all patients were gathered in EDTA tubes and stored at − 20 °C for less than 2 months. A TIANamp Genomic DNA Kit (TianGen, Beijing, China) was used to extract genomic DNA from whole blood before analysis. Genotyping for SNP screening analyses was conducted using the KASP (kompetitive allele specific PCR) assay. Equal amounts of genomic DNA (0.8 μl/patient) from DR and DNR patients were mixed with the KASP Master Mix and KASP Assay Mix. Next, the SNP-containing DNA fragments were amplified by PCR. The PCR program was as follows: initial denaturation at 94 °C for 15 min, 10 cycles of denaturation at 94 °C for 20 s and annealing at 65 °C (0.8 °C decrease every cycle) for 1 min; and 27 cycles of a final extension at 59 °C for 1 min. Primers for the KASP SNP assays were designed using Primer Premier 5.0, and allele frequencies were analysed using IntelliQube software (LGC Genomics, UK).

### Statistical analyses

Continuous data are shown as the mean ± SD. Categorical variables are reported as numbers (percentages) or percentages. The normality of the distribution of quantitative variables was verified by the Kolmogorov-Smirnov test. After the normal distribution test, the comparison of continuous variables among groups of diabetic subjects was made by ANOVA for normally distributed variables. The *χ*^*2*^ test was used to compare categorical variables. Gene counting was applied to determine allele frequencies, and the *χ*^*2*^ test was used to verify departures from Hardy-Weinberg equilibrium. The comparison between allele and genotype frequencies was made among groups of subjects using the *χ*^*2*^ test. *SPSS* (version 20.0; *SPSS*, Inc., Chicago, IL, USA) was employed to conduct statistical analyses. We considered *P* values less than 0.2 to be great for inclusion in multivariate analysis. Multivariate analysis, adjusting for the use of insulin treatment, systolic blood pressure and glomerular filtration rate (GFR), was performed with binary logistic regression analysis. According to the outcomes, odds ratios and 95% confidence intervals (CIs) are shown. We considered *P* values less than 0.05 to be statistically significant.

## Results

### Clinical data of the research population

Table 1 summarizes the features of the subjects. The cases and controls were 58.5 and 58.95 years of age on average, respectively, and the groups included 44.04 and 44.87% females, respectively, indicating good match between two groups with regard to age and sex (both *P* > 0.05). No significant variation was noted in the duration of DM, body mass index, HbA1c, HDL cholesterol, total cholesterol or LDL cholesterol between the groups. There were significant differences in analyzing systolic blood pressure and using insulin treatment and glomerular filtration rate (GFR) between the groups.

**Table 1:** Comparison of clinical features between two groups

Clinical characteristics (Variable)	NDR group (***n*** = 386)	PDR group (***n*** = 316)	***P-value***
Female [n (%)]	170 (44.04)	140 (44.87)	0.877
Age [years, mean ± SD]	58.50 ± 11.44	58.95 ± 10.16	0.699
Duration of DM [years, mean ± SD]	9.53 ± 7.07	10.10 ± 7.24	0.472
Body mass index (BMI) [kg/m^2^, mean ± SD]	23.922 ± 3.127	24.701 ± 4.760	0.093
HbA1c [% (mmol/mol), mean ± SD]	8.668 ± 2.432	8.677 ± 2.520	0.976
Insulin treatment [n (%)]	180 (46.63)	212 (67.52)	**0.001**
Hypertension [n (%)]	168 (43.52)	142 (45.22)	0.750
Systolic blood pressure [mmHg, mean ± SD]	130.65 ± 17.41	135.05 ± 18.06	**0.025**
Diastolic blood pressure [mmHg, mean ± SD]	81.01 ± 9.56	80.17 ± 10.40	0.437
Total cholesterol [mM, mean ± SD]	4.290 ± 1.188	4.448 ± 1.258	0.259
HDL cholesterol [mM, mean ± SD]	1.028 ± 0.273	1.080 ± 0.360	0.155
LDL cholesterol [mM, mean ± SD]	2.711 ± 0.967	2.853 ± 0.908	0.190
Glomerular filtration rate (GFR) [ml/min, mean ± SD]	84.970 ± 33.218	75.951 ± 28.971	**0.013**

Bold indicates statistically significant results

### Candidate gene and single nucleotide polymorphism selection

We selected 4 SNPs (rs1800896, rs2010963, rs2070600, rs2910164), which from IL-10, VEGF, RAGE and miR-146a, respectively. And these genes were related to DR in at least one population or are logical candidate genes according to the present understanding of the pathogenesis of DR; there are selected SNPs in the promoter areas, 5′ UTR regions, or coding areas of candidate genes. In addition, the total call rates of these 4 SNPs (rs1800896, rs2010963, rs2070600, and rs2910164) were 99.43, 98.86, 97.72, and 84.90%, respectively.

### Polymorphisms of 4 SNPs in type 2 diabetic subjects based on the presence of PDR

The distribution of the genotype and allele frequencies of the 4 SNPs’ polymorphisms in type 2 diabetic subjects based on the presence of PDR are shown in Table 2. All 4 SNPs were distributed in accordance with Hardy-Weinberg equilibrium. No significant variation in the genotype and allele frequencies of the miR-146a polymorphism (rs2910164) was noted between subjects with PDR and those without DR, indicating that this SNP might not be related to the presence of PDR. However, the data show that the other 3 SNP (rs1800896, rs2010963 and rs2070600) were significantly associated with the presence of PDR.

**Table 2:** Analysis of 4 SNPs in NDR and PDR groups

Gene	SNP	Allele	NDR [%]	PDR [%]	Geno-type	NDR [n (%)]	PDR [n (%)]	χ^2^ test	Logistic regression analysis	
								*P-value*	odds ratio (95% CI)	*P*_*adjust*_*-value*
IL-10	rs1800896	T	95.85	90.71	TC	32 (8.29)	50 (16.03)	**0.002**	2.366 (1.144, 4.894)	**0.020**
		C	4.15	9.29	CC	0 (0)	4 (1.28)	**0.026**	1.524 (0)	0.999
					TT	354 (91.71)	258 (82.69)	**0.001**	Reference	
					Total	386	312			
VEGFA	rs2010963	C	39.00	34.62	CG	214 (56.02)	140 (44.87)	**0.003**	0.588 (0.366, 0.946)	**0.028**
		G	60.99	65.38	CC	42 (10.99)	38 (12.18)	0.627	1.277 (0.578, 2.821)	0.546
					GG	126 (32.98)	134 (42.95)	**0.007**	Reference	
					Total	382	312			
RAGE	rs2070600	C	72.80	82.00	CC	200 (51.81)	200 (66.67)	**0.001**	Reference	
		T	27.20	18.00	TT	24 (6.22)	8 (2.67)	**0.029**	0.180 (0.037, 0.872)	**0.033**
					CT	162 (41.97)	92 (30.67)	**0.002**	0.448 (0.266, 0.753)	**0.002**
					Total	386	300			
miR-146a	rs2910164	C	63.68	65.88	CC	158 (41.58)	132 (44.59)	0.432	–	
		G	36.32	34.12	GG	54 (14.21)	38 (12.84)	0.606	–	
					CG	168 (44.21)	126 (42.57)	0.669	–	
					Total	380	296			

Bold indicates statistically significant results

*P*_*adjust*_ values were adjusted for systolic blood pressure and using insulin treatment and glomerular filtration rate (GFR)

Multivariable logistic regression analysis showed that 3 SNPs (rs1800896, rs2010963 and rs2070600) were associated with the PDR phenotype after adjustment for insulin therapy, systolic blood pressure and GFR. The frequency of the TC genotype of rs1800896 was higher among subjects with PDR than in those without DR (16.03% vs. 8.29%, *P* = 0.002). The frequency of the TT genotype was lower in subjects with PDR (82.69% vs. 91.71%, *P* = 0.001). After multivariable analysis, the TC genotype was determined to be related to an increased risk of PDR. The odds ratio (OR) was 2.366, with a 95% confidence interval (CI) ranging from 1.144 to 4.894 (*P*_*adjusted*_ = 0.020). The frequency of the CG genotype of rs2010963 was reduced in subjects with PDR compared with those without DR (44.87% vs. 56.02%, *P* = 0.003). After multivariable analysis, the CG genotypes were related to a decreased risk of PDR. The OR of the CG genotype was 0.588, with a 95% CI ranging from 0.366 to 0.946 (*P*_*adjusted*_ = 0.028). The frequency of the CC genotype of rs2070600 was increased among subjects with PDR than in those without DR (66.67% vs. 51.81%, *P* = 0.001). The frequency of the CT genotype was lower in subjects with PDR (30.67% vs. 41.97%, *P* = 0.002). Multivariable analysis revealed that the other 2 genotypes (TT and CT) were related to a reduced risk of PDR. The OR of the TT genotype was 0.180, with a 95% CI ranging from 0.037 to 0.872 (*P*_*adjusted*_ = 0.033). The OR of the CT genotype was 0.448, with a 95% CI ranging from 0.266 to 0.753 (*P*_*adjusted*_ = 0.002).

## Discussion

Many studies have suggested that genetic factors are important in the context of DR. In this study, we analyzed the association of 4 SNPs selected from 4 DR-associated factors in an independent cohort of patients in Guangxi Province with type 2 diabetes mellitus (T2DM). Our data showed significant associations with the IL-10, VEGF and RAGE genes.

It has been proposed that DR is associated with persistent low-grade inflammation [30]. Interleukin-10 (IL-10) prevents the generation of proinflammatory cytokines and stimulates the proliferation, differentiation and survival of several types of immune cells [31]. Most cells of the adaptive and innate immune systems such as dendritic cells, leukocytes, and macrophages express IL-10 [31]. DR progression may be promoted by IL-10, an anti-inflammatory cytokine with strong deactivating nature [14]. IL-10 gene rs1800896 polymorphism (IL-10 -1082G/A polymorphism) in the promoter region was associated with production of IL-10 [32]. And it is reported that the IL-10 gene polymorphism is related to the risk of DR among various populations [14, 33, 34]. This study showed that the TC genotype was associated with the risk for PDR. The various genetic backgrounds of the study samples, sample sizes, exposure to environmental factors, and clinical phenotypes of PDR may explain the conflicting outcomes reported by the abovementioned studies. According to the multivariable analyses, the IL-10 rs1800896 polymorphism is associated with a significant risk of PDR. However, these outcomes should be explained carefully due to the limited sample sizes in the stratified analyses and the limited power. However, evidence for a possible effect between the rs1800896 polymorphism and several T2DM risk elements is indicated by our findings.

Vascular endothelial growth factor (VEGF) drives angiogenesis, breaks down the blood-retinal barrier, excites the growth of endothelial cells, induces neovascularization, and enhances vascular permeability in the ischaemic retina [35, 36]. Increased expression level of VEGF has been observed in DR patients [37]. In Yang’s study [38], they summarized that the rs2010963 was a risk contributor to PDR in overall populations, while no significant association was detected between rs2010963 and PDR risk in Caucasians. Our analysis demonstrated that carriers of the CG genotype had a lower risk for PDR compared with those with the GG genotype. In accordance with our study, Awata and coworkers [39] found no association between the CC genotype of rs2010963 polymorphism and PDR. Carriers of another 2 additional homozygous genotypes exhibited altered susceptibility to DR, suggesting that rs2010963 might be an important genetic marker for DR among patients in Guangxi Province with T2DM. Many factors could determine the differences in the findings, such as sample size, study design, and sunlight exposure [40].

Receptor for advanced glycation end product (RAGE) gene polymorphisms impact DR due to pathophysiological information related to retinopathy and advanced glycation end products (AGEs) [6]. The RAGE gene is located on the short arm of chromosome 6: 6p21.3 [17]. AGEs result from the nonenzymatic glycation of proteins and lipids [41]. AGEs are observed at enhanced levels in individuals with diabetes and can result in enhanced oxidative stress and receptor-mediated activation and secretion of different cytokines [41]. The RAGE polymorphism assessed in this study occurs at a predicted N-linked glycosylation motif in the AGE binding site, impacting AGE-RAGE interactions [6]. This study analysed the RAGE SNP (rs2070600), and the results showed that TT genotype or T allele carriers were associated with a reduced risk for PDR. Similar strong relationships between rs2070600 and diabetic retinopathy were also observed in Asian Indians and Asian Chinese people with type 2 diabetes and in an Indian study [6, 17]. Moreover, analysis of the allelic frequency of rs2070600 in different ethnic groups showed different results. The T allele frequency in this research was 18.00% in the PDR group, which is similar to the findings of an earlier report in the Chinese population (23.1%) [42] and another report in the Japanese population (17.3%) [43]. In previous reports, the T allele occurred with an incidence of 5% in Caucasians [44] and 2% in Indians [45]. Allelic variants of the RAGE gene may alter protein function and gene expression, which may influence disease progression. The high proportion of variant alleles in the Chinese population may confer enhanced susceptibility to diabetic side effects in this population.

In Kaidonis’ study [16], rs2910164 was found to potentially enhance susceptibility to retinal injury via a pathway involved in both angiogenesis and breakdown of the blood retinal barrier. This SNP significantly related to DN in patients suffering from type 1 diabetes mellitus (T1DM) after multivariate analysis [16]. In our study, we collected samples from T2DM patients to analyse the risk of DR, but T1DM and T2DM are unique diseases with various aetiologies. DR progression is impacted by environmental elements that may occur under the background of a given type of diabetes mellitus. Furthermore, DR commonly develops early in susceptible patients with T1DM [46]. Statistical analysis revealed that rs2910164 was not significantly related to DR. Further studies with a larger cohort size are warranted to more accurately assess these given phenotypes in relation to microRNA-146a (miR-146a) SNPs.

Potential limitations of the current study should be taken into account. First, the sample size was not large, which may have caused our study to be underpowered. Second, we cannot exclude confounding effects of unmeasured variables that may affect the stability of blood glucose levels, such as dietary and other lifestyle factors. Third, no detailed information regarding DR severity or treatment response was obtained, which limited our conclusions. Fourth, we planned to avoid population substructures in our research. Nevertheless, it is possible that the positive and negative outcomes obtained in this study may be attributed to subtle population stratification, and the results should be considered suggestive instead of definitive. Moreover, our study requires a more direct assessment of the association between SNPs and related serum levels. The mechanisms underlying these SNPs in DR merit further study.

## Conclusion

According to the outcomes of this research, the rs1800896 polymorphisms in the IL-10 gene, rs2010963 in the VEGFA gene and rs2070600 in the RAGE gene are related to the risk of PDR in the Han Chinese population of Guangxi Province. Our findings provide suggestive evidence that these polymorphisms may be involved in the pathogenesis of PDR and should be examined further. Moreover, our study suggests that the rs2910164 polymorphism in the miR-146a gene may not be related to DR in the Guangxi Province population. Nevertheless, these findings should be examined by additional well-designed multicentre studies with larger sample sizes that include gene–environment interaction assessments.

## Data Availability

The datasets of primer used during the current study are available online (https://pan.baidu.com/s/1V45I_EnSPxPzrV768sGw6w, code: f06e) or from the corresponding author on reasonable request.

## Abbreviations

DR: Diabetic retinopathy
PDR: Proliferative diabetic retinopathy
T2DM: Type 2 diabetes mellitus
T1DM: Type 1 diabetes mellitus
KASP: Kompetitive allele specific PCR
DNA: Deoxyribonucleic acid
GWAS: Genome-wide association study
GFR: Glomerular filtration rate
IL-10: Lnterleukin-10
VEGF: Vascular endothelial growth factor
RAGE: Receptor for advanced glycation end product
